# Management of pregnant and postnatal women with pre-existing diabetes or cardiac disease using multi-disciplinary team models of care: a systematic review

**DOI:** 10.1186/s12884-014-0428-5

**Published:** 2014-12-20

**Authors:** Debra Bick, Sarah Beake, Lucy Chappell, Khaled M Ismail, David R McCance, James SA Green, Cath Taylor

**Affiliations:** King’s College London, Florence Nightingale Faculty of Nursing and Midwifery, James Clerk Maxwell Building, 57 Waterloo Road, London, SE1 8WA UK; Women’s Health Academic Centre, Guys and St Thomas’ NHS Foundation Trust, 10th floor North Wing, St Thomas’ Hospital, Westminster Bridge Road, London, SE1 7EH UK; Birmingham Centre for Women’s and Children’s Health, School of Clinical & Experimental Medicine, College of Medical & Dental Sciences, University of Birmingham, Birmingham, B15 2TT UK; Regional Centre for Endocrinology and Diabetes, Royal Victoria Hospital, Grosvenor Road, Belfast, BT12 6BA UK; Whipps Cross Hospital, Barts NHS Trust, London, UK; Department of Health and Social Care, London South Bank University, London, UK

**Keywords:** Multidisciplinary team, Pregnancy, Diabetes, Cardiac disease, Maternity, Antenatal, Postnatal, Complex pregnancies

## Abstract

**Background:**

More women with an increased risk of poor pregnancy outcome due to pre-existing medical conditions are becoming pregnant. Although clinical care provided through multi-disciplinary team (MDT) working is recommended, little is known about the structure or working practices of different MDT models, their impact on maternal and infant outcomes or healthcare resources. The objectives of this review were to consider relevant international evidence to determine the most appropriate MDT models of care to manage complex medical conditions during and after pregnancy, with a specific focus on pre-existing diabetes or cardiac disease in high income country settings.

**Methods:**

Quantitative and qualitative evidence of MDT models of care for the management of pregnant/postnatal women with pre-existing diabetes and cardiac disease was considered. A search of the literature published between January 2002 - January 2014 was undertaken. Methodological quality was assessed using checklists developed by the Joanna Briggs Institute. Given limited primary and secondary research evidence, guidelines and opinion papers were included. Two independent reviewers conducted critical appraisal of included papers.

**Results:**

Nineteen papers were included from UK, Canada, USA, the Netherlands and Singapore. No studies were found which had compared MDT models for pregnant/postnatal women with pre-existing diabetes or cardiac disease. Two small retrospective studies reported better outcomes for women with cardiac disease if an MDT approach was used, although evidence to support this was limited. Due to study heterogeneity it was not possible to meta-analyse data. No evidence was identified of MDT management in the postnatal period or impacts of MDT working on healthcare resources.

**Conclusions:**

Despite widespread promotion of MDT models of care for pregnant and postnatal women with pre-existing diabetes or cardiac disease, there is a dearth of primary evidence to inform structure or working practices or beneficial impact on maternal and infant outcomes or healthcare resources. Primary research into if or how MDT models of care improve outcomes for women with complex pregnancies is urgently needed.

**Electronic supplementary material:**

The online version of this article (doi:10.1186/s12884-014-0428-5) contains supplementary material, which is available to authorized users.

## Background

Adverse maternal outcomes in high income countries are increasing [1]. As more women with an increased risk of poor pregnancy outcome due to pre-existing medical conditions are becoming pregnant [2,3], the importance of care to minimise adverse outcomes is clear. Two conditions of particular concern are pre-existing diabetes and cardiac disease. Diabetes is the most common pre-existing medical condition to complicate pregnancy in the United Kingdom (UK) with approximately 2% to 5% of pregnant women having pre-existing diabetes [4,5]. Coronary heart disease affects 0.2% - 4% of pregnant women in western industrialized countries [6,7].

Women with pre-existing diabetes or cardiac disease are classed as ‘high risk’ due to an increased risk of poorer outcomes for them or for their infants as a consequence of their health circumstances. For infants, these include a higher risk of fetal malformation, premature birth, high or low infant birth weight, admission to neonatal intensive care, and in some cases infant death [3-5,8]. For women with diabetes, there is a higher risk of caesarean section, miscarriage, pregnancy induced hypertension and pre-eclampsia [9,10]. For women with cardiac disease, depending on cardiac condition diagnosed, there is a higher risk of acute heart failure, arrhythmias, and in rare cases, maternal death most commonly from cadiomyopathy and pulmonary hypertension [8,11,12].

It has been advocated that many adverse outcomes could potentially be avoided with appropriate and timely identification and communication of the risk or problem, effective team working between appropriate specialists and promotion of seamless care across health sector boundaries [5,13,14]. Overall perinatal mortality rates for women with diabetes cared for by an MDT antenatally in regional centres in Northern Ireland were reported to be marginally better than among women with diabetes treated at local centres in one retrospective study which examined 10 years of data [15]. However, high level evidence to support benefits and a clear description of what constitutes an optimal MDT model is lacking. An enquiry into maternity service provision for women with diabetes in England, Wales and Northern Ireland found that women with diabetes continued to have significantly increased risks of adverse pregnancy outcomes compared to the general maternity population [5]. This was despite the 1989 St. Vincent’s Declaration which included a five year target for women with diabetes to achieve similar pregnancy outcomes to women without diabetes through better medical management, pre-conception care and pregnancy monitoring [16]. A recent review of 12 population studies published in the last decade with data on over 14,000 women with type 1 diabetes and over four million women from the background population assessed the impact of the Declaration. The prevalence of four fetal and neonatal complications were compared, which were two to five times higher in women with type 1 diabetes than in the general population indicating that pregnancy outcomes for women with diabetes had not improved [17].

In the UK, antenatal guidance from the National Institute for Health and Care Excellence (NICE, previously the National Institute for Health and Clinical Excellence [18]) includes recommendations that women who need ‘additional care’, including those with cardiac disease and endocrine disorders should be identified at their antenatal booking appointment. However, as NICE antenatal guidance [18] is for routine care of healthy women, the ‘additional care’ women with pre-existing cardiac disease or diabetes should be offered is not described. Although guidance is available to support management for some conditions or circumstances known to increase the risk of poor pregnancy outcome, for example hypertension in pregnancy [19], lack of agreed guidelines to ensure such women are appropriately managed by the clinical team or how their care should be organized may limit the potential benefit on maternal and/or infant outcomes. Furthermore, the structure and role of the MDT in maternity care is not addressed, with reference made in existing guidance limited to ensuring consultations are based on the individual needs of the woman and her baby [18].

Numerous high profile reports in the UK have recognized the need to improve team-working between maternity care professionals to improve safety and experience of maternity services among women with high risk pregnancies [13,14,20], but have failed to describe how healthcare providers (in community and hospital based settings) and clinicians (for example, midwives, obstetricians, GPs, cardiologists, diabetologists) should work together or how teams should be structured. Furthermore, evaluations of such models of care are lacking. Models of MDT working exist in some UK maternity services for some women with pre-existing medical conditions, for example diabetes [5] and systemic lupus erythmatosus [21] but the impact of these teams on maternal and infant outcomes is yet to be established, with little consistency in models of care women can access within and between maternity organizations (C Taylor, personal communication). In cancer care the introduction of tumour-specific referral pathways and “hub and spoke” models of local and specialist MDTs appear to have overcome shortfalls in the quality, equity and safety of care [22]. MDTs have now been recommended in the UK for other conditions including diabetes, stroke and neurological conditions, chronic obstructive disease and coronary heart disease [22] and could be an appropriate approach for optimal management of women with complex medical conditions during and after pregnancy.

In the absence of consistent guidance or recommendations for the management of women with pre-existing medical complications, the aim of this review was to assess the evidence to determine the most appropriate organizational models of care to manage pre-existing diabetes and cardiac disease during and after pregnancy in high income country settings.

## Methods

The review was developed using the process described by The Joanna Briggs Institute (JBI) to consider and appraise all forms of available evidence relevant to optimal MDT models of pregnancy and postnatal care in high income country settings, as defined by the World Development Indicators [23], focusing on pre-existing diabetes and cardiac disease. The JBI is an international research and development organisation whose strength is in facilitating systematic reviews of research that use a range of methodologies, including qualitative methods, economic and policy research (www.joannabriggs.org). The JBI levels of evidence are shown in below: 

The Joanna Briggs Institute levels of evidence

- Level 1 (strongest evidence) Meta-analysis (with homogeneity) of experimental studies (e.g. RCT with concealed randomization) OR one or more large experimental studies with narrow confidence intervals;
- Level 2 One or more smaller RCTs with wider confidence intervals OR Quasi-experimental studies (without randomization);
- Level 3 a. Cohort studies (with control group); b. Case-controlled; c. Observational studies (without control group);
- Level 4 Expert opinion, or physiology bench research, or consensus

(for further details, see Additional file 1)

To examine the evidence regarding MDT models of care and determine which outcomes MDT models are associated with, specific review questions were developed:

### Primary questions

- What models of *management* of pre-existing diabetes or cardiac disease in pregnant/postnatal women have been evaluated and what outcomes were included in these evaluations?

### Secondary questions

- What models to prompt appropriate and timely *referral* of pregnant/postnatal women with pre-existing diabetes and cardiac disease have been evaluated and what outcomes are they associated with?
- What are the barriers to timely identification of pre-existing diabetes and cardiac disease in pregnant/postnatal woman and how might these be overcome?
- Has use of information and communication technology (ICT) supported decision making when caring for pregnant/postnatal women with pre-existing diabetes and cardiac disease?
- What are the potential economic costs and benefits of MDTs to meet the needs of pregnant/postnatal women who have pre-existing diabetes and cardiac disease?

The literature search focused on the identification of quantitative and qualitative evidence on organisational models of care for pregnant/postnatal women with pre-existing diabetes and cardiac disease. As it was anticipated that there would be limited primary research evidence the search also included non-research publications, including national and international guidelines and opinion papers. Together with describing any models of care found (e.g. membership structure, process, referral pathways, working practices) we searched for evidence regarding the relationship between MDTs and the following processes and/or outcomes in line with a protocol developed and agreed by all authors prior to commencing the review:

- Timing and method of initial referral and management of pregnant women with pre-existing diabetes or cardiac disease
- Maternal mortality, physical and psychological morbidity
- Infant mortality, physical and psychological morbidity
- Women’s experiences of MDTs in maternity care
- Health professionals’ experiences of MDTs in maternity care
- The use of technology in maternity MDTs
- Economic outcomes of maternity MDTs

A search strategy was developed to identify papers, restricted to those published in English. A four-step strategy was utilised. In the first stage optimal search terms were identified using CINAHL and MEDLINE. Key words and subject headings were then searched using the following databases from January 2002 to February 2013: CINAHL, MEDLINE, Cochrane Library, EMBASE, Maternal & Infant Care, Scopus and PsycINFO. Inclusion of papers published from January 2002 onwards was selected to inform contemporaneous models of care for these two conditions, the increasing numbers of women with poor pregnancy outcomes related to these conditions highlighted in UK national Confidential Enquiries into maternal deaths since 2001 [24] and publication of recommendations for management of women with complex pregnancies in policy and guidelines [14]. The third stage involved searching the reference list of identified papers for additional papers. A fourth stage included a search of policy and grey literature, for example, dissertations and theses, conference proceedings or relevant professional organization reports (for example the Royal College of Obstetricians & Gynaecologists’ guidance on cardiac disease[3]) with recommendations for practice. The search strategy used for MEDLINE is included in Additional file 2 as an example of subject headings and limits used in searches. The searches were re-run in January 2014 and no new papers relevant to the aims of the review were identified. Studies were excluded if they focused on gestational diabetes, if care described did not include obstetric care or input from multidisciplinary teams or if the study had been undertaken in a middle or low income country.

Identified papers were initially assessed for relevance based on title by one reviewer (SB) and then further assessed using the abstract by two of the authors (SB, CT). Following the initial evaluation of the abstracts, these two reviewers (CT, SB) independently applied study inclusion/exclusion criteria and assessment of methodological quality using standardised critical appraisal instruments developed by the JBI (www.joannabriggs.org). Any disagreements that arose between the reviewers were resolved through discussion with a third reviewer (DB). Critical appraisal instruments developed by the JBI were used appropriate to the study methods, with risk of bias highlighted within the assessment. As it was anticipated that there would be few relevant randomised controlled trials, evidence from studies which used other quantitative methods as well as evidence from qualitative studies, policy and opinion papers were considered.

## Results

**Figure 1:**
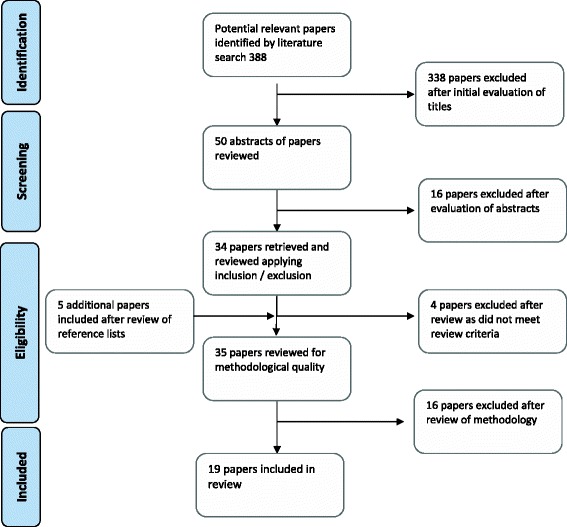
**Flow chart of stages of searching.**

A total of 388 papers were identified from the initial search and all titles assessed, with 50 papers initially considered relevant to the aims of the review (Figure 1). The abstracts of these papers were independently assessed by SB and CT to see if they met review inclusion criteria.

The full texts of 34 papers were retrieved and assessed further to confirm whether they met inclusion criteria and a further four papers excluded. The reference lists of the retrieved papers were searched for possible further relevant papers and five additional papers included at this point. The remaining papers were appraised using relevant appraisal checklists for the type of study under consideration, following which 16 papers were excluded. Reasons for excluding these papers included (for example) lack of congruity between the research methodology and methods used to collect the data, study aims not clearly defined, poor description of outcomes and lack of appropriate analysis. A total of 19 papers were included in the review including one Cochrane review, two primary research papers, ten opinion pieces and six national guideline and consensus papers. All referred to care in high income country settings. Four papers referred to women with diabetes and 15 to women with cardiac disease. As no randomised controlled trials (RCTs) or appropriate quantitative studies were identified, data could not be statistically combined for a meta-analysis. Data which were available were synthesised into a narrative summary. Table 1 presents findings on the level of evidence presented following appraisal of included studies using JBI criteria.

**Table 1:** **Papers included in systematic review of MDTs for management of pre-existing diabetes or cardiac disease**

**Authors**	**Design/JBI level of evidence**	**Cardiac or T1, T2 diabetes**	**Summary data**
Abdin S. (2006) [25] (UK)	Opinion paper Level 4	Cardiac	Experience of a specialist tertiary referral unit and recommendations for organisational management.
Arafeh J.M. and Baird S.M. (2006) [26] (USA)	Opinion paper Level 4	Cardiac	Review of the management of women with cardiac disease throughout pregnancy.
Confidential Enquiry into Maternal and Child Health. (2007) [5] (UK)	National enquiry Level 3	Diabetes	Findings of a national enquiry on pregnancy in women with type 1 & 2 diabetes (excluding gestational diabetes) in England, Wales & Northern Ireland which included; a survey of diabetes maternity services of women with type 1 & 2 diabetes, a descriptive study of 3830 pregnancies, a national confidential enquiry reviewing demographic, social and lifestyle factors, and clinical care in 422 pregnancies.
Curtis S.L. et al. (2009) [27] (UK)	Retrospective study Level 3	Cardiac	Describes experience at one tertiary referral unit of management of heart disease in pregnancy, including; adverse events, adherence to guidelines, and areas of suboptimal management. 177 pregnancies in 155 women were included.
Dob D.P. and Yentis S.M. (2006) [28] (UK)	Opinion paper Level 4	Cardiac	Obstetric anaesthetist guide to the management of pregnant women with congenital heart disease and own experience in a tertiary referral unit over 10 years.
Greutmann M.K. et al. (2010) [29] (UK)	Retrospective study Level 3	Cardiac	Retrospective cohort study of the outcomes 0f 76 pregnancies in 47 women, with congenital heart disease and residual haemodynamic right outflow tract lesions, attending one tertiary referral unit.
Herrey A. and Nelson-Piercy C. (2010) [30] (UK)	Opinion paper Level 4	Cardiac	Review of the management of women with cardiac disease throughout pregnancy.
Kafka H. et al. (2006). [31] (UK & Canadian)	Opinion paper Level 4	Cardiac	Review of the management of women with cardiac disease throughout pregnancy, including; the effects of pregnancy on the circulation system, the risks and care of the woman and an appendix describing the team approach.
McElduff A. et al. (2005) [32] (Australian)	National guidance/consensus opinion Level 4	Diabetes	The Australasian Diabetes in Pregnancy Society consensus guidelines for the management of type 1 & 2 diabetes in relation to pregnancy.
National Institute for health and Clinical Excellence (2008) [9] (UK)	National guidance Level 3	Diabetes	Management of diabetes and its complications from pre-conception to the postnatal period
Pieper P.G. (2012) [33] (Netherlands)	Opinion paper Level 4	Cardiac	Review of the management of women with cardiac disease throughout pregnancy and delivery. Illustrates complications that can arise unexpectedly.
Ray P. et al. (2004) [34] (UK)	Opinion paper Level 4	Cardiac	Review of the most common causes of cardiac disease and the management of women with cardiac disease in pregnancy.
Steer et al. [41]	Consensus statement Level 4	Cardiac	Consensus views arising from a study group; Heart Disease and pregnancy including; antenatal, intrapartum and postnatal care.
Royal College of Obstetricians & Gynaecologists. (2011) [3] (UK)	Professional body guidance Level 4	Cardiac	Cardiac disease and pregnancy, Good Practice guidance to provide a summary of expert opinion on the general principles of the management of cardiac disease pre-conception, antenatally, intrapartum and postnatally.
Roberts R. and Ketchell A. (2012) [35] (UK)	Opinion paper Level 4	Cardiac	Review the assessment, management and care of women with cardiac problems, with a focus on those who have or develop mitral valve stenosis in pregnancy.
Tan JY-L. (2010) [36] (Singapore)	Opinion paper Level 4	Cardiac	Review of the management of women with cardiac disease throughout pregnancy.
The European Society of Cardiology (ESC) (2011) [7]	Guidelines Level 4	Cardiac	ESC Guidelines on the management of cardiovascular diseases during pregnancy. Members of the task force are selected experts in the field from across Europe.
Tieu J. et al. (2011) [37] (Australia)	Cochrane review Level 3	Diabetes	Cochrane review of preconception care for the diabetic women for improving maternal and infant health. The review included one trial (involving 53 women) which did not report on the pre-specified outcomes of the review.
Uebing A. et al. (2006) [38] (UK)	Opinion paper Level 4	Cardiac	Review of the management of women with cardiac disease throughout pregnancy, birth and postnatally and the risks.

Many of the excluded papers were opinion pieces that only briefly mentioned MDTs in conclusion as a recommendation for practice, with little or no mention of organisational management or content of care for women with pre-existing diabetes or cardiac disease or any of the other outcomes of interest. Apart from two small retrospective cohort studies from the UK (one study which assessed outcomes of women with cardiac disease who received MDT care in pregnancy [29]; and a second study which assessed if management guidelines were followed for women with cardiac disease [27]), no other primary research papers were identified. Given the dearth of primary research papers, papers that included an overview of organisational management or national guidance were included. Ten opinion papers on the management of cardiac disease were included [25,26,28,30,31,33-36,38]. No opinion papers were identified which referred to the management of pre-existing diabetes. Six opinion papers were written by authors based in the UK, one paper included authors from the UK and Canada, one the USA, one the Netherlands and one from Singapore. For the most part these opinion papers were primarily describing the *medical* management of cardiac disease but also included a section on the *organisational* management of cardiac disease.

A Cochrane review [37] of preconception care for diabetic women (based on one trial involving 53 women) which did not report on pre-specified areas of interest for the current review, was included, as was the Confidential Enquiry into Maternal and Child Health national enquiry into diabetes in pregnancy[5] and NICE guidance[9] on management of diabetes in pregnancy. The CEMACH enquiry presented findings from three programmes of work: a survey of diabetes maternity services for women with type 1 and type 2 diabetes in England, Wales and Northern Ireland [39]; a descriptive study of 3808 pregnancies among women with type 1 and type 2 diabetes in the same UK countries, identified at booking between 2002 and 2003 with follow up to 28 days post-birth [40]; and a national confidential enquiry including a case control analysis with cases selected from the descriptive study sample, to review demographic, social and lifestyle factors and clinical care in 442 pregnancies to women with type 1 and type 2 diabetes and association with pregnancy outcome [5]. A paper describing the national Australian consensus guidance on diabetes in pregnancy [32] was also included. National guidance and consensus papers for cardiology included the RCOG good practice guideline [3] and study group statement [41]and the European Society of Cardiology (ESC) guidelines on the management of cardiac disease in pregnancy [7]. The following sections present the findings relevant to each of the primary and secondary review questions.

### Primary questions

#### What models of management of pre-existing diabetes and cardiac disease in pregnant/postnatal women have been evaluated and what outcomes are they associated with?

No studies were identified which had evaluated models of management of pregnant or postnatal women with the conditions of interest. Studies which were included only referred to cardiac disease, and only to management and pregnancy outcome in single site settings. These provided a low level of evidence (level 3) and risk of bias from use of retrospective designs. None of the studies referred to women’s experiences of care or impact on their psychological and other aspects of their physical health and well-being or health professionals’ experiences of MDT care.

#### Cardiac disease

One retrospective cohort study of the outcomes of 76 pregnancies which continued beyond 24 weeks gestation in 47 women with congenital heart disease and residual haemodynamic right outflow tract lesions [29] included women who attended a joint cardiology/obstetric clinic in one tertiary referral centre in London. This was referred to by the authors as a ‘specialist MDT’ (p1765) and included a cardiologist, an obstetrician, an anaesthetist, a haematologist and a clinical nurse specialist. All women were seen by a Grown Up Congenital Heart disease (GUCH) cardiologist at 14–16 weeks gestation, with follow up during pregnancy planned on an individual basis depending on complexity and risk. A detailed labour and birth plan was developed for all women following discussion with the MDT at 32–34 weeks gestation. Details of which members of the MDT were involved in the follow up of women after 14–16 weeks gestation, or how the team worked together (e.g. whether they had formal meetings, and if these included the women as well) were not provided.

The authors described maternal and fetal outcomes as ‘good’ [29]. Obstetric complications among the 76 pregnancies included two women with systemic hypertension, two with pre-eclampsia, four with cervical insufficiency, two with postpartum haemorrhage, two with retained placental products and one with cholestasis of pregnancy. Seven women developed complications including right sided heart failure (RHF) at a mean of 26.9 weeks gestation which was identified and treated with no major adverse events. Information on whether this was identified more quickly due to MDT involvement was not provided, although the authors referred to ‘prompt’ intervention improving signs and symptoms of RHF in 5 of the women. Neonatal outcomes in pregnancies that continued beyond 24 weeks included two stillbirths and 13 premature births. Four infants were born with congenital heart disease.

Although not a pre-specified aim of the study, the authors compared their outcomes with seven other studies of comparable patient groups. Some differences were noted in outcomes, which may have been due to chance or differences in patient baseline characteristics. A smaller number of women with severe pulmonary regurgitation (PR) were found compared to the index study, perhaps reflecting different recommendations about contraindications of pregnancy among women with moderate to severe PR in different healthcare settings and population groups.

Abdin [25] described experiences of providing cardiac care to women in the same London unit as Greutmann et al. [29] but from a midwifery perspective. If women’s cardiac conditions were considered to be moderately or highly complex, the risk of complications during pregnancy were reviewed at a joint obstetric/cardiac meeting and a plan of care formulated, involving the pregnant woman and her family. A monthly ‘link’ meeting was held with neonatologists and obstetricians to discuss maternal and neonatal management of women but details of who else could be involved in these meetings (including the woman) were not provided. Abdin [25] suggested that the approach of the joint clinic helped reduce women’s anxiety and provided continuity of care by the midwifery staff although evidence to support this statement was not presented.

Curtis et al. [27] undertook a retrospective study of 177 pregnancies in 155 women with cardiac disease who attended a high risk pregnancy clinic in a tertiary referral centre in the South West of England to assess if care provided met standards derived from an amalgamation of guidelines including the European Society of Cardiology (ESC) consensus opinion document [42] and CEMACH [24], both of which included recommendations about MDT care. No details of the current MDT model of care implemented at the unit were described. Data were reviewed from 1999 to 2005, and over half of the women had pre-existing cardiac disease. Mean pregnancy gestation at clinic referral was 22 weeks.

The authors concluded that pregnancies among women with cardiac disease were increasing and for the most part recommended management standards were met, although suboptimal MDT management in some cases was identified when compared against guideline recommendations. These included no provision of a MDT approach involving obstetricians and anaesthetists for five women with congenital heart disease (the cases identified were all prior to 2004), lack of pre-conception advice and inadequate post-partum follow-up - only 97 (55%) of women were seen in an out-patient clinic within six months of giving birth. In 138 (78%) cases, MDT discussion was ‘not indicated’ but it was unclear as to whether this was because information was not documented or a discussion was not needed. No detail of what MDT discussion entailed was provided. The authors stressed that the majority of women in their cohort had benign symptoms and signs of cardiac disease which may not have required specialist clinic assessment. However as they postulate, assessment of what is ‘benign’ could be misconstrued outside of a specialist clinic assessment.

### Secondary questions

#### What models to prompt appropriate and timely referral of pregnant/postnatal women with pre-existing diabetes and cardiac disease have been evaluated and what outcomes are they associated with?

No studies were identified which specifically evaluated models of appropriate or timely *referral* of pregnant or postnatal women with pre-existing diabetes and cardiac disease or the outcomes they were associated with. Papers which made some reference to *identification* of women with complex medical needs (cardiac disease and diabetes) were included (level 4 evidence).

#### Cardiac disease

The importance of women with pre-existing cardiac disease being reviewed pre-conception in a pre-pregnancy clinic or by their cardiologist [7,26,27] and need for pre-pregnancy counselling [3,7,28,29,31,33,36,41] was mentioned in several papers which considered clinical management of these women, much of which was based on expert opinion, and no papers cited empirical evidence to support this. Some authors advocated that planning for pregnancy should commence in adolescence although evidence to support this or what planning should include was not provided [3,28].

Once pregnancy was confirmed, guideline recommendations referred to the need for all women with a heart murmur or history of cardiac defect to have a risk assessment undertaken by a MDT [3,7,41]. For example, the RCOG Good Practice Guidance [3] recommended that as there are so many types of cardiac disease often with different implications for care and potential outcome, women should be seen in early pregnancy at a joint MDT clinic attended by a consultant obstetrician, cardiologist and anaesthetist. A further MDT meeting was recommended at around 32–34 weeks gestation to discuss plans for the birth and postpartum management [3]. Evidence to support these recommendations was not provided.

All included papers recommended that following an initial risk assessment, women assessed to be at low risk could be returned to routine antenatal care [3,27,31,33,38]. The RCOG 2006 consensus group statement on heart disease and pregnancy [41] recommended direct self-referral to clinical teams should be enabled for women with heart disease, to reduce potential bureaucratic delays. Women assessed as having moderate or high risk should be referred to a tertiary centre/high risk team, although input from members of the team could vary depending on risk [7,31,33,34,38]. The RCOG (2006) consensus group statement [41] also suggested that initial risk assessment should determine the frequency and content of antenatal care. No papers defined risk, or provided evidence to support a consensus on this. Table 2 presents modified risk criteria from WHO included in the ESC guidelines published in 2011. Other authors’ derived risk criteria from previously published clinical data sets, for example Arafeh and Baird [26] based criteria for low or minimal risk, intermediate or moderate risk, or high or major risk using data from a previously published clinical study of women in pregnancy [43], a textbook chapter [44] and the New York Heart Association classification for grade of risk (reference not provided by the authors).

**Table 2:** **Modified WHO classification of maternal cardiovascular risk: principles (ESC 2011)**

**Risk class**	**Risk of pregnancy by medical condition**
I	No detectable increased risk of maternal mortality and no/mild increase in morbidity.
II	Small increased risk of maternal mortality or moderate increase in morbidity.
III	Significantly increased risk of maternal mortality or severe morbidity. Expert counselling required. If pregnancy is decided upon, intensive specialist cardiac and obstetric monitoring needed throughout pregnancy, childbirth, and the puerperium.
IV	Extremely high risk of maternal mortality or severe morbidity; pregnancy contraindicated. If pregnancy occurs termination should be discussed. If pregnancy continues, care as for class III.

The clinician responsible for referring women to the high risk clinic also varied in the included papers, indicating that in some cases women were seen by a generic ‘gate-keeper’ who would refer the woman to a high risk pregnancy clinic. Curtis et al. [27] reported that 43% of referrals in their audit were made by obstetricians, 25% by general practitioners (family doctors), congenital or general cardiology clinicians referred around 10% of women respectively, with a small number of women self referring. Method of referral was unknown in 10% of women.

#### Diabetes

Pre-pregnancy planning was considered in three papers for women with pre-existing diabetes. The Cochrane review of pre-conception care for diabetic women [37] reported evidence of benefit was equivocal, based on one small trial with 53 women, and recommended need for further large trials. The CEMACH national enquiry into diabetes in pregnancy [5] highlighted the importance of women having access to a pre-conception service with a MDT to minimise the risk of fetal malformation. It recommended that MDTs should include as a minimum, an obstetrician, diabetes physician, diabetes specialist nurse, diabetes midwife and dietician. Of the 442 women whose cases were reviewed by the enquiry, 28% had attended for preconception care in an adult diabetes clinic, 15% with their GP and 26% in a hospital-based MDT clinic but it was unclear if this included a maternity component. No information on pre-conception care was available for just under a third of cases reviewed. The enquiry panel assessors found that 73% of 267 women (87% of 133 cases and 60% of 134 controls) had received suboptimal preconception care, defined by panel assessors as lack of: pre-conception advice, contraceptive advice, provision of higher dose folic acid, appropriate screening and management of diabetes complications and MDT involvement. Women who received suboptimal preconception care were more likely to have a poor pregnancy outcome (OR 5.2, 95% CI 2.7 - 10.1, adjusted for maternal age and deprivation) with additional case–control analysis showing an association with fetal congenital anomaly [5].

The NICE guidance for diabetes in pregnancy [9] and the Australasian Diabetes in Pregnancy Society consensus guidelines for the management of type 1 and type 2 diabetes in pregnancy [32] also recommended that women planning pregnancy should be offered pre-conception care and advice to raise awareness of potential problems. NICE diabetes guidance [9] recommended a structured education programme, with pregnant women offered immediate contact with a joint diabetic and antenatal clinic but did not define the structure or membership of such a clinic or how often it should meet. McElduff et al. [32] cited the meta analysis by Ray et al. [45] reporting a significantly lower prevalence of major congenital abnormalities in the babies of diabetic women who attended pre-pregnancy counselling, compared to those who did not attend (Relative Risk 0.36; 95% CI, 0.22–0.59; absolute risk, 2.1% v 6.5%).

The CEMACH national enquiry into diabetes in pregnancy [5] recommended that specialist MDTs should provide regular education days for primary and secondary care health professionals involved in management of women with diabetes, to cover preconception, pregnancy and postnatal care. No evidence of the benefit of this approach was provided.

#### What are the barriers to timely identification of pre-existing diabetes and cardiac disease in pregnant/postnatal woman and how might these be overcome?

High level evidence to address this question was not identified. Papers included provided comment or opinion for women with cardiac disease and diabetes (Level 4 evidence, Table 1).

#### Cardiac disease

A number of potential barriers were described. Kafka [31] in an opinion paper suggested that unplanned pregnancies for women with cardiac disease could be life threatening, which was a particular concern for teenagers who have a high unplanned pregnancy rate and raised the need for counselling for younger women to be implemented before they reached the adult heart clinic by a paediatric cardiologist. Kafka [31] also discussed the feasibility of having all relevant specialists in one clinic at the same time, and suggested it was important that this happened at least at the first antenatal visit so women could get an understanding of the different roles of the clinicians involved in their care. The paper also identified questions raised by clinicians including whether high-risk clinics should be a long distance from a woman’s home. The author’s response was that benefits outweighed the inconvenience of women having to travel.

Curtis et al. [27] reported that 18% of women who were pregnant had no pre-conception counselling documented and those having their first baby were generally young with a median age of 22 years. Improvements to services made following the study included placing an ink stamp in the notes of women of childbearing age attending adult cardiac disease clinics to remind cardiologists to offer pregnancy advice and need to highlight the possibility of inheritance of cardiac disease.

Ray et al. [34] suggested in a narrative review of the most common causes of cardiac disease and the management of women in the UK with cardiac disease in pregnancy, that women at greatest risk of suboptimal care were recent immigrants to the UK and those in lower socio economic groups. They considered that within some ethnic groups the desire to have children could lead to under-reporting of cardiac symptoms but provided no evidence to support this. The authors suggested that language difficulties and late presentation to antenatal care were also barriers to better pregnancy outcomes, citing the findings of the 2001 CEMD report chapter on cardiac disease [24]. The provision of appropriate referral to specialist centres and timely support from the MDT, which was not defined in the paper, could minimize the consequences of poorly controlled heart disease in pregnancy [34].

Several opinion papers highlighted the failure of an accurate diagnosis of cardiac problem or recognition of the severity of the problem by attending health professionals but provided no further detail on issues for clinical skills and competencies [26,34,36].

#### Diabetes

Possible barriers to timely identification of women with pre-existing diabetes were reported by the CEMACH national enquiry [5]. Less than a fifth of maternity units in England, Wales and Northern Ireland provided structured multidisciplinary preconception care for women with type 1 or type 2 diabetes based on findings from the CEMACH national survey [39]. Furthermore the enquiry noted poor documentation of pre-pregnancy counselling [5]. With respect to care in pregnancy, communication deficiencies between clinical disciplines were noted. Of 338 women whose notes were reviewed, the notes of 222 (56%) women were identified as indicating communication problems between *health professionals*. With specific reference to problems within the MDT, a lack of dedicated joint clinics and poor sharing of information between obstetricians and diabetes physicians was identified. Poor communication between health professionals *and women* was noted in 169 (47%) of 360 women for whom notes were available with particular reference to lack of discussion about plans for care during pregnancy. The enquiry assessors also highlighted that in around 10% of these women, social and lifestyle issues, including non-attendance at planned appointments, unplanned pregnancies, language difficulties, difficult domestic circumstances and erratic or busy lifestyles also contributed to poorer pregnancy outcomes.

#### How has using information and communication technology (ICT) supported decision making when caring for pregnant/postnatal women with pre-existing diabetes and cardiac disease?

No primary or secondary research studies were identified which had specifically evaluated the use of information and communication technology. None of the included opinion papers, guidelines or consensus papers referred to this issue.

#### What are the potential economic costs and benefits of multidisciplinary teams to meet the needs of pregnant/postnatal women who have pre-existing diabetes and cardiac disease?

No primary or secondary research studies were identified which had evaluated the potential economic costs and benefits of MDTs, and no references were made to these areas in the included opinion papers or guidelines.

## Discussion

A systematic review was undertaken to assess the evidence regarding multi-disciplinary models of care for women with pre-existing diabetes and cardiac disease during and after pregnancy in high income country settings. Nineteen papers from a range of countries were included. Much of the evidence identified was low in the JBI evidence hierarchy (level 3 or 4) with potential risk of bias from use of retrospective designs, reliance on expert opinion and focused on pre-conception or clinical management during pregnancy. Due to the limited evidence identified, it was only possible to present a narrative review of study findings. Despite national and international policy and guideline recommendations for MDT management for women with pre-existing diabetes or cardiac disease, no evaluations of different structures or working practices of MDT teams or impact on maternal or infant outcomes were identified, with limited or no evidence identified to inform any of the areas of interest of this review.

High risk pregnancies among women with pre-existing diabetes or cardiac disease were selected as the primary focus for the review for several reasons; there is a high prevalence of pre-existing diabetes in pregnancy; recommendations for MDT management have been available for some time; and more women with diabetes are becoming pregnant; maternal adverse outcomes from cardiac disease are high and medical issues are complex [5,6,8]. As both conditions are also associated with adverse perinatal outcomes, they are medical complications of importance to women, obstetricians, midwives, and primary and secondary care physicians. As such there was an expectation that they would have been the focus of research into MDT management, however this was not the case. Despite pre-existing diabetes being one of the most commonly experienced complex medical problems in pregnancy with national and international guidance to support MDT management and provision of care [9,32], only four included papers referred to the structure and working practices of teams caring for women with diabetes. Despite being a complex medical problem with a high risk of adverse outcome, evidence to support MDT management of women with pre-existing cardiac disease was found in only 15 papers which met the review inclusion criteria.

Studies which referred to MDT models of care supported the value of these for women with pre-existing diabetes or cardiac disease, and need for all relevant clinicians to be involved in women’s care, despite not providing evidence of evaluation or justification for the structure or processes of such care. Only two primary research studies were identified which were small, from single site studies, focused on cardiac disease and provided retrospective data with a high risk of bias. The two studies were from UK-based teams but described different models of care for women with cardiac disease [27,29]. It was unclear if the models described were standard within these centres, how often the MDT members met, if meetings took place without the woman being present, how communication about a woman’s case was shared between the members of the team or how team ‘hierarchies’ operated. Levels of communication across primary and secondary care sectors and with other maternity providers were not described. These are important areas to address if audit and benchmarking of clinical outcomes are to be undertaken at local or national level to assess the benefit of current MDT models or if outcomes could be enhanced through development of an optimal MDT model of management.

Where guidelines for MDT care were available, audit of one single site setting showed that guidelines were either not followed and/or care provided was not documented [27]. Curtis et al. [27] who audited care for women at one maternity unit against ESC and other guidelines, found that their MDT model (which was not described in detail) for women with congenital cardiac disease was not always followed. The study by Greutmann et al. [29] which aimed to evaluate pregnancy outcome and risk factors for adverse events among women with cardiac disease, was the only one which considered the composition of a specialist MDT and evaluated its role in planning care for women from 14–16 weeks of pregnancy. The authors attempted to compare their outcomes with previous studies which had published data on pregnancy outcomes, several of which were from non UK settings. While comparable outcomes were generally good with no maternal deaths reported, differences in patient populations studied, MDT management of pregnancy complications and pre-pregnancy interventions suggests findings should be treated with caution.

No studies were identified which had specifically evaluated MDT models of care to prompt timely and appropriate referral of pregnant women with complex medical needs, although some papers referred to the need for risk assessment among women with pre-existing cardiac disease, and some views that younger women should have pregnancy planning raised with them. Similarly, for women with diabetes, pre-pregnancy planning was proposed as important to prevent pregnancy complications, however evidence to support how and when these services should be offered and to whom, or how risks of pregnancy could be communicated to women and their families was lacking. Evidence from the 2007 CEMACH diabetes enquiry showed a very small proportion of units in England, Wales and Northern Ireland provided structured multidisciplinary preconception care for women with pre-existing diabetes, and noted poor documentation of pre-pregnancy counseling. The importance of timely referral for care was referred to in several papers, as was the need to consider aspects of the woman’s lifestyle. Postnatal care and issues relevant to support longer term maternal and infant health outcomes, for example, support for breastfeeding or follow up by the MDT team, were not addressed in any studies reviewed nor was evidence of impact on women’s experiences of their care or health professionals experiences of providing care.

Potential barriers to effective management of women with the medical conditions of interest included late booking in pregnancy, language barriers, and non-attendance at appointments, although it was unclear how widespread these issues were, with around 10% of women identified in the CEMACH enquiry [46]. No studies had considered use of information and communication technology to support decision making, or extent to which women were involved in any decisions about their care, despite Department of Health policy [47] on need for greater patient engagement in all decisions about their care. Given the implications for health service resources of care for women with complex medical conditions during and after pregnancy, and for the health of their infants, there is a clear gap in evidence of what an optimal MDT model of care should comprise, and the clinical and cost effectiveness of different organisational models of care. Of concern is that currently, women with pre-existing diabetes or cardiac disease could be managed differently within and between units, informed or not informed by ‘best practice’ guidance, resulting in inequity of outcomes for the woman and her infant.

In addition to the limited evidence of models of MDT and impacts of MDT management on processes and outcomes of interest, the review found no evidence of what an optimal MDT model should include. If suggestions for MDT membership were made in any of the papers reviewed, this was based on assumption that this would be the most appropriate team composition, with no evidence to support how teams should be structured, operationalized on a day to day basis or how its structure and organisation impact on outcomes evaluated.

A recent large prospective mixed methods study of 12 MDTs (for different chronic medical conditions) in general population groups in London and the North Thames region of England [48] which collected data from meetings, interviews with 53 MDT members and over 2,000 patient records concluded that for the different disease groups not all team meetings necessarily resulted in more effective decision making. MDT benefits could not be assumed for every chronic condition. The researchers also found that the addition of more professional groups to the MDT led to a reduction in implementation of treatment plans for some disease groups, with adjusted odds of implementation reduced by 25% for each additional professional group represented at the MDT meeting. Having a good ‘team climate’ as measured using a team measure inventory and team skill mix positively impacted on the implementation of treatment plans. A review of evidence regarding team effectiveness in healthcare teams suggested that factors at individual (staff/patient characteristics), team (structure and processes) and organisational (structure, rewards, training) levels should be considered [49].

Boon and colleagues [50] developed a conceptual framework to support the description, comparison and evaluation of team-based health care practices in Western health systems. Their framework identifies seven different models of team-oriented practice ranging on a continuum from ‘parallel’ at one end of the continuum (defined as a model of team care characterised by independent health clinicians working in a common setting) to ‘integrative’ at the other end (defined as comprising among other facets, an interdisciplinary team approach guided by consensus building and shared vision of health enabling each clinician and patient to contribute their particular knowledge and skills within the context of a shared, synergistically charged plan of care). Application of this classification to the current diverse models of MDT working for pregnant and postnatal women and examining relationships with process (timeliness of decisions) and outcomes for the woman and her infant would be extremely valuable to support decisions regarding the organisation of care for high risk pregnancies.

## Conclusion

Despite national and international clinical policy, guideline and consensus support for potential benefits of MDT based organisational models of care for women with pre-existing diabetes or cardiac disease who become pregnant, there is a dearth of evidence to support optimal MDT structure and working practices, or if current MDT models of care have a beneficial impact on maternal and infant outcomes and healthcare resources. The only primary evidence identified indicated that care is unlikely to be standardised within or between units, with women likely to receive a range of approaches to management which may or may not be informed by evidence. It is not possible from current evidence to recommend what an optimal organisational model of care should include or if an MDT model approach promotes highest quality care leading to better maternal and infant outcomes, women’s experiences of care, or use of healthcare resources. Given the paucity of evidence currently available, primary research to consider clinical and cost effectiveness outcomes of MDT approaches to organising maternity care for pregnant and postnatal women with these conditions is urgently needed.

### Ethical approvals

Ethics approval was not required for this study.

## Additional files

Additional file 1:
**JBI levels of evidence.**
Additional file 2:
**Example of MEDLINE search strategy for diabetes.**
