# Supplementary material for: Management of pregnant and postnatal women with pre-existing diabetes or cardiac disease using multi-disciplinary team models of care: a systematic review
Source: BMC Pregnancy Childbirth. 2014 Dec 20;14:428. doi: 10.1186/s12884-014-0428-5 (PMC4296678; doi:10.1186/s12884-014-0428-5)
Supplement: Additional file 1: — JBI levels of evidence. [file 12884_2014_428_MOESM1_ESM.docx]

**Additional file 1: Table S1; JBI Levels of Evidence**

| Levels of  Evidence | Feasibility  F (1-4) | Appropriateness  A (1-4) | Meaningfulness  M (1-4) | Effectiveness  E (1-4) | Economic Evidence  EE (1-4) |
| --- | --- | --- | --- | --- | --- |
| 1 | Meta-synthesis  of research with  unequivocal  synthesised  findings | Meta-synthesis  of research with  unequivocal  synthesised  findings | Meta-synthesis  of research with  unequivocal  synthesised  findings | Meta-analysis  (with homogeneity)  of experimental  studies (eg RCT  with concealed  randomisation)  OR One or more  large experimental  studies with  narrow confidence  intervals | Meta-synthesis  (with homogeneity)  of evaluations of  important alternative  interventions  comparing all clinically  relevant outcomes  against appropriate  cost measurement,  and including a  clinically sensible  sensitivity analysis |
| 2 | Meta-synthesis  of research  with credible  synthesised  findings | Meta-synthesis  of research  with credible  synthesised  findings | Meta-synthesis  of research  with credible  synthesised  findings | One or more  smaller RCTs with  wider confidence  intervals  OR Quasi-experimental studies (without  randomisation) | Evaluations of  important alternative  interventions  comparing all clinically  relevant outcomes  against appropriate  cost measurement,  and including a  clinically sensible  sensitivity analysis |
| 3 | a. Meta-synthesis  of text/opinion  with credible  synthesised  findings  b. One or more  single research  studies of high  quality | a. Meta-synthesis  of text/opinion  with credible  synthesised  findings  b. One or more  single research  studies of high  quality | a. Meta-synthesis  of text/opinion  with credible  synthesised  findings  b. One or more  single research  studies of high  quality | a. Cohort studies  (with control  group)  b. Case-controlled  c. Observational  studies (without  control group) | Evaluations of  important alternative  interventions  comparing a limited  number of appropriate  cost measurement,  without a clinically  sensible sensitivity  analysis |
| 4 | Expert opinion | Expert opinion | Expert opinion | Expert opinion,  or physiology  bench research, or  consensus | Expert opinion, or  based on economic  theory |
