# Supplementary material for: Management of pregnant and postnatal women with pre-existing diabetes or cardiac disease using multi-disciplinary team models of care: a systematic review
Source: BMC Pregnancy Childbirth. 2014 Dec 20;14:428. doi: 10.1186/s12884-014-0428-5 (PMC4296678; doi:10.1186/s12884-014-0428-5)
Supplement: Additional file 2: — Example of MEDLINE search strategy for diabetes. [file 12884_2014_428_MOESM2_ESM.docx]

**Additional file 2. Example of search strategy (MEDLINE)**

MDT 1 – Medline for diabetes (total 25 hits)

1. mutidisciplinary.mp.

2. limit 1 to (english language and humans and yr="2002 -Current")

3. Patient Care Team/

4. limit 3 to (english language and humans and yr="2002 -Current")

5. group visits.mp.

6. limit 5 to (english language and humans and yr="2002 -Current")

7. 2 or 4 or 6

8. Maternal Health Services/ or Prenatal Care/ or maternity.mp. or Pregnancy/

9. limit 8 to (english language and humans and yr="2002 -Current")

10. Obstetrics/

11. limit 10 to (english language and humans and yr="2002 -Current")

12. Pregnancy/ or Prenatal Care/ or antenatal care.mp.

13. limit 12 to (english language and humans and yr="2002 -Current")

14. Perinatal Care/

15. limit 14 to (english language and humans and yr="2002 -Current")

16. Postnatal Care/

17. limit 16 to (english language and humans and yr="2002 -Current")

18. Patient Care/

19. limit 18 to (english language and humans and yr="2002 -Current")

20. 9 or 11 or 13 or 15 or 17 or 19

21. 7 and 20

22. Diabetes Mellitus, Type 1/ or diabetes.mp. or Diabetes Mellitus, Type 2/ or Diabetes Mellitus/

23. limit 22 to (english language and humans and yr="2002 -Current")

24. 21 and 23

MDT 2b – Medline for cardiac disease (total 30 hits)

1. mutidisciplinary.mp.

2. limit 1 to (english language and humans and yr="2002 -Current")

3. Patient Care Team/

4. limit 3 to (english language and humans and yr="2002 -Current")

5. group visits.mp.

6. limit 5 to (english language and humans and yr="2002 -Current")

7. 2 or 4 or 6

8. Maternal Health Services/ or Prenatal Care/ or maternity.mp. or Pregnancy/

9. limit 8 to (english language and humans and yr="2002 -Current")

10. Obstetrics/

11. limit 10 to (english language and humans and yr="2002 -Current")

12. Pregnancy/ or Prenatal Care/ or antenatal care.mp.

13. limit 12 to (english language and humans and yr="2002 -Current")

14. Perinatal Care/

15. limit 14 to (english language and humans and yr="2002 -Current")

16. Postnatal Care/

17. limit 16 to (english language and humans and yr="2002 -Current")

18. Patient Care/

19. limit 18 to (english language and humans and yr="2002 -Current")

20. 9 or 11 or 13 or 15 or 17 or 19

21. 7 and 20

22. heart disease.mp. or Heart Diseases/

23. limit 22 to (english language and humans and yr="2002 -Current")

24. Coronary Disease/

25. limit 24 to (english language and humans and yr="2002 -Current")

26. Cardiovascular Diseases/

27. limit 26 to (english language and humans and yr="2002 -Current")

28. congenital heart disease.mp.

29. limit 28 to (english language and humans and yr="2002 -Current")

30. Heart Defects, Congenital/ or heart defects.mp.

31. limit 30 to (english language and humans and yr="2002 -Current")

32. 23 or 25 or 27 or 29 or 31

33. 7 and 20 and 32

34. from 33 keep 10-12,15,17-18

35. multidisciplinary team.mp.

36. limit 35 to (english language and humans and yr="2002 -Current")

37. 7 or 36

38. 20 and 32 and 37
